# The Digital Skills, Experiences and Attitudes of the Northern Ireland Social Care Workforce Toward Technology for Learning and Development: Survey Study

**DOI:** 10.2196/15936

**Published:** 2020-09-23

**Authors:** Jonathan Synnott, Mairead Harkin, Brenda Horgan, Andre McKeown, David Hamilton, Declan McAllister, Claire Trainor, Chris Nugent

**Affiliations:** 1 School of Computing University of Ulster Newtownabbey United Kingdom; 2 Northern Ireland Social Care Council Belfast United Kingdom

**Keywords:** social work, learning, teaching methods, surveys, health care workers, mobile phone, digital divide, distance education, educational technology

## Abstract

**Background:**

Continual development of the social care workforce is a key element in improving outcomes for the users of social care services. As the delivery of social care services continues to benefit from innovation in assistive technologies, it is important that the digital capabilities of the social care workforce are aligned. Policy makers have highlighted the importance of using technology to support workforce learning and development, and the need to ensure that the workforce has the necessary digital skills to fully benefit from such provisions.

**Objective:**

This study aims to identify the digital capability of the social care workforce in Northern Ireland and to explore the workforce’s appetite for and barriers to using technology for learning and development. This study is designed to answer the following research questions: (1) What is the digital capability of the social care workforce in Northern Ireland? (2) What is the workforce’s appetite to participate in digital learning and development? and (3) If there are barriers to the uptake of technology for learning and development, what are these barriers?

**Methods:**

A survey was created and distributed to the Northern Ireland social care workforce. This survey collected data on 127 metrics that described demographics, basic digital skills, technology confidence and access, factors that influence learning and development, experience with digital learning solutions, and perceived value and challenges of using technology for learning.

**Results:**

The survey was opened from December 13, 2018, to January 18, 2019. A total of 775 survey respondents completed the survey. The results indicated a workforce with a high level of self-reported basic digital skills and confidence. Face-to-face delivery of learning is still the most common method of accessing learning, which was used by 83.7% (649/775) of the respondents; however, this is closely followed by digital learning, which was used by 79.0% (612/775) of the respondents. There was a negative correlation between age and digital skills (r_s_=−0.262; *P*<.001), and a positive correlation between technology confidence and digital skills (r*_s_*=0.482; *P*<.001). There was also a negative correlation between age and the perceived value of technology (r_s_=−0.088; *P*=.02). The results indicated a predominantly motivated workforce in which a sizable portion is already engaged in informal digital learning. The results indicated that lower self-reported basic digital skills and confidence were associated with less interest in engaging with e-learning tools and that a portion of the workforce would benefit from additional basic digital skills training.

**Conclusions:**

These promising results provide a positive outlook for the potential of digital learning and development within the social care workforce. The findings provide clear areas of focus for the future use of technology for learning and development of the social care workforce and considerations to maximize engagement with such approaches.

## Introduction

### Background

The Northern Ireland Social Care Council (Social Care Council) is the regulatory body for the social care workforce in Northern Ireland. Established in 2001, the Social Care Council is one of the 12 health and social care regulators within the United Kingdom. The Social Care Council has over 42,000 registered members comprising social care workers and managers, social workers, and social work students. The purpose of the Social Care Council is to ensure that health and social care workers are regulated against relevant laws and standards [1].

Continual development of the social care workforce, in the form of postregistration training and learning, is a key element in enabling better outcomes for the users of social care services, as highlighted in the Social Care Council’s 2017-2021 corporate plan [2]. This continual development is also a requirement to maintain the Social Care Council registration. The UK Department of Health and Social Care has released the Learning and Improvement Strategy for Social Workers and Social Care Workers 2019-2027 [3]. Within this strategy document, priority 6 focuses on social care practice within the digital world. In particular, this priority highlights the need to improve e-learning methodology and ensure that the workforce has the necessary skills to make the best use of the available technology. In 2017, Kennedy and Yaldren [4] stated that digital literacy was increasingly becoming a key requirement in contemporary health care and health education. They detailed several areas of health education that could benefit from technology-enhanced learning. These included accessibility and inclusivity, flexibility, development of professional identities and behaviors, signposting of resources, and improved collaboration. A report released by Health Education England in 2017 [5] also highlights the need for digital skills within the health and social care sector, emphasizing that the health care sector has traditionally been slow to adopt new digital tools and technologies. The report states that modern health and social care environments require lifelong, self-directed learners, which can be facilitated through digital tools. The report also highlights how an increase in digital literacy can dramatically increase the uptake and adoption of new digital tools and technologies, ultimately increasing the quality of care provided. The report highlights several key challenges in increasing the digital capabilities of the health and social care staff. One of these key factors focuses on human behaviors and attitudes toward digital literacy, including lack of confidence and unwillingness to use technology, and barriers in terms of organizational policy or lack of investment in technology.

### Previous Work

In 2017, the Digital Health & Care Institute [6] published results obtained from a survey of 539 members of the social care workforce. This survey collected information on the workforce’s attitudes toward digital technology and digital skills issues. This research highlighted that the social care staff and social care managers were aware of the potential benefits of digital technology in providing care services. However, the majority of the managers who responded to the survey stated that they believed the lack of staff capability was a challenge for using digital technology. This was in contrast to the opinion of the staff respondents, of which over 90% said that they were confident or very confident in their basic digital skills.

In 2019, De Gagne et al [7] reviewed the application of microlearning within health professional education in which knowledge or skills are acquired in the form of small units for continuing education. The review discussed the facilitation of microlearning through technology-based solutions, including podcasts and social media. This educational approach has been found to have a positive effect in areas such as knowledge and confidence in various practice areas. Wilkinson and Ashcroft [8] further highlighted the potential benefits of social media for health professional education, including the ability to overcome geographical and time barriers, and the fact that many students already access these platforms as part of their daily routine.

In 2014, a workforce learning strategy was developed by the Skills for Care and Development, Sector Skills Council [9]. This strategy highlighted the need for new learning resources to be developed around mobile technologies and stated that the workforce would require a level of digital literacy. As this 5-year strategy ended in 2019, this provides an opportunity to assess the current state of the workforce and identify opportunities for future direction. The use of mobile apps to educate the social care workforce is at an early stage [10]. Nevertheless, the Social Care Council has demonstrated previous success in the launch of digitally enabled learning solutions, including the Domiciliary Care Toolkit [11] and a series of award-winning Understanding Child Development apps that were updated in 2018 [10,12].

### Objectives

The Social Care Council is currently developing a new learning and development strategy that will focus on the use of technology-enabled learning and development. This paper summarizes the results of a collaboration between Ulster University and the Social Care Council. The collaboration aimed to investigate the digital capability of the social care workforce in Northern Ireland and the attitudes of the workforce toward digital learning and development solutions. The purpose of this study is to identify the readiness of the workforce to engage with such digital solutions and to identify the potential barriers to the uptake that could then be addressed early in the design process.

This study is designed to answer the following research questions:

1. What is the digital capability of the regulated social care workforce in Northern Ireland?

2. What is the workforce’s appetite to participate in digital learning and development?

3. If there are barriers to the uptake of technology for learning and development, what are these barriers?

## Methods

### Distribution

A survey was developed to answer these research questions. This survey was hosted on SurveyMonkey [13] and a link to the survey was distributed to the registered social care workforce via email. A participant information sheet was also distributed along with the survey link. The participant information sheet highlighted that participation would take 10 min, data would be stored on a secure Ulster University server for 10 years, the purpose of the study, that participation was voluntary, and contact details of the principle investigator.

The survey was further publicized through the Social Care Council website and social media accounts. To encourage participation, respondents were entered into a prize draw for a tablet computer and for 1 of 5 £50 (US $65.75) gift vouchers. The gift vouchers were sponsored by Silverbear PLC [14]. The anonymity of responses was maintained by collecting the participant contact details in a separate survey to the main data collection survey. The Ulster University Research Ethics Filter Committee reviewed and approved the study on December 11, 2018. The link to the survey was distributed from December 13, 2018, and the survey website remained open for data collection until January 18, 2019.

### Design

The survey was used to collect both quantitative and qualitative data. The questions facilitated the collection of categorical and ordinal responses in the form of multiple-choice questions. Respondents were also offered the opportunity to provide qualitative, free text responses to elaborate on response selection where appropriate. In total, the survey facilitated the collection of 127 metrics for analysis, which were split into 2 sections. Each section was displayed on a separate page. Respondents were able to review any responses until the point of submission. Responses to all closed-ended questions were mandatory, and responses to any open-ended question were optional. Participation and view rates were not calculated, as unique internet protocol addresses were not logged as part of the ethical approval to maintain anonymity.

Section 1 collected demographic information, including job role, area of practice, age, and gender. This section also collected information relating to digital skills, confidence, and the frequency of using technology. Information regarding digital skills was captured through responses to a series of 10 statements, each regarding a technology-based skill, such as finding a previously visited website and installing apps. For each statement, respondents were asked to state whether they could perform this task if they were asked to. These statements were adapted from The Tech Partnership’s *Get Digital: Basic Skills Assessment* questionnaire, which was featured in Lloyds Bank’s UK Consumer Digital Index 2018 [15]. Reuse permission was granted.

Section 2 focused on attitudes and experiences with the use of digital technology to support learning and development at work. Respondents were asked about factors that influence them to learn and develop and the methods, location, and frequency of their learning and development. In addition, respondents were asked how useful they had found existing tools for digital learning and development and whether they would be interested in engaging with digitally enabled learning and development at home, at their workplace, or not at all. Finally, respondents were asked about their level of agreement with 6 statements regarding the value of technology to support learning and development and 7 statements regarding the challenges associated with technology to support learning and development. To maintain the logical flow of the survey, the items were not randomized.

The survey was reviewed by an independent sample of computing researchers and social care workers. These reviews primarily investigated the clarity of the questions, appropriateness of the closed-ended question response options, and length of the survey. Feedback from these users were discussed among the research team and agreed amendments were incorporated into the final version.

The inclusion criterion for the study was the membership of the Social Care Council’s registered workforce. There were no exclusion criteria. This facilitated a convenience sampling of the target population. This was an open survey; however, only members of the registered Social Care Council workforce were given the participation URL.

The use of a web-based survey was the most cost-effective method to maximize exposure to a large number of potential respondents. The recruitment of participants through digital channels was identified as a potential source of bias within the study by potentially targeting members of the workforce who are already digitally active. However, all members of the workforce are encouraged to renew their Social Care Council registration on an annual basis using the Social Care Council’s web-based registration portal. In addition, hardcopies of the survey questionnaires were offered upon request. Therefore, it can be argued that this web-based approach would not disadvantage or omit any member of the workforce from participating and that the bias associated with the study should be minimal.

## Results

### Overview

The survey received responses from 959 respondents. Of these, 19.2% (184/959) were removed from the analysis of the results because of partial completion. A total of 775 (80.8%) fully completed survey responses were included in the analysis of the results. No hardcopies of the survey questionnaires were requested. Table 1 provides an overview of the job role and gender of the respondents, and Table 2 provides an overview of the age distribution of the respondents.

Of the 539 social care workers, 31.2% (n=168) were domestic care workers, 29.3% (n=158) were residential care workers, 26.0% (n=140) were supported living care workers, and 13.5% (n=73) were daycare workers. Of the 222 respondents in the social work setting (excluding social work students), the most common sector of practice was health and social care trust (n=162, 73.0%) followed by the voluntary sector (n=23, 10.4%). Other common sectors of practice included the education sector (n=12, 5.4%) and the justice sector (n=9, 4.1%). The most common social work settings were mental health and addiction (n=23, 10.4%), training, education and governance (n=22, 9.9%), and looked-after children (n=19, 8.6%).

There was a substantially higher number of responses from females (629/775, 81.2%) than that of males (136/775, 17.5%). This imbalance reflects the gender imbalance of the Social Care Council’s overall registered workforce. As of October 2019, 45,255 members of the registered workforce consisted of 86.14% (n=38,983) females and 13.70% (n=6204) males.

**Table 1:** Overview of the respondent job role and gender.

Job role	Gender				Overall, n (%)
	Female, n (%)	Male, n (%)	Other, n (%)	Prefer not to say, n (%)	
Social care worker	442 (82.0)	92 (17.1)	2 (0.4)	3 (0.6)	539 (69.5)
Social worker	175 (78.8)	42 (18.9)	0 (0.0)	5 (2.3)	222 (28.6)
Social work student	12 (85.7)	2 (14.3)	0 (0.0)	0 (0.0)	14 (1.8)

**Table 2:** Overview of the age distribution of the respondents.

Age category (years)	Number of respondents, n (%)
15-24	61 (7.9)
25-44	332 (42.8)
45-64	371 (47.9)
≥65	7 (0.9)
Prefer not to say	4 (0.5)

### Digital Skills

Table 3 provides an overview of the digital skills results received from the respondents in each job role. Overall, the skills with the largest deficit included “Solve a problem with a device or digital service using online help,” with 101/775 (13.0%) respondents stating that they could not do this if asked to; “Check that information you found online is accurate,” with 70/775 (9.0%) respondents indicating that they could not do this if asked to; and “Buy and install apps on a device,” with 7.1% (55/775) respondents indicating that they could not do this if asked to.

A digital skills score was calculated, which provided an overall summary of each respondent’s digital skills based on responses to each of the 10 skills statements. Table 4 provides an overview of the mean digital skills score calculated for each job role. Cronbach α for the 10 digital skills score items was .877. The Kruskal–Wallis test indicated no significant difference (*P*=.08) between the social care worker and social worker digital skills score. A high mean digital skills score indicates a general high level of digital skills capabilities.

The relationship between age and digital skills was explored. Of note, responses under the age category of “Prefer not to say” have been excluded. Table 5 provides an overview of the mean digital skills score obtained for each age group.

It can be seen that there is a general trend of digital skills score decline with age. The Kruskal–Wallis test confirmed that there was a significant difference (*P*<.001) in the digital skills score between the age groups. There was a weak negative correlation between age group and digital skills score (r_s_=−0.262; *P*<.001).

**Table 3:** Digital skills responses versus job role.

Response	Job role			Overall, n (%)
	Social care worker, n (%)	Social worker, n (%)	Social work student, n (%)	
I could do this if I was asked to	5098 (94.6)	2103 (94.7)	139 (99.3)	7340 (94.7)
I couldn’t do this if I was asked to	242 (4.5)	113 (5.1)	1 (0.7)	356 (4.6)
I have no idea what you are talking about	50 (0.9)	4 (0.2)	0 (0.0)	54 (0.7)

**Table 4:** Mean digital skills score versus job role.

Job role	Digital skills score^a^, mean (SD)
Social care worker	9.46 (1.47)
Social worker	9.47 (1.10)
Social work student	9.93 (0.27)
Overall	9.47 (1.36)

^a^The maximum possible digital skills score is 10.

**Table 5:** An overview of mean digital skills score versus age group.

Age group (years)	Digital skills score, mean (SD)
15-24	9.84 (0.55)
25-44	9.73 (1.17)
45-64	9.20 (1.50)
≥65	7.86 (3.02)

### Confidence

Respondents were asked to provide an indication of their confidence with using 4 types of technologies: smartphones, tablets, desktop computers, and laptops. Confidence with each technology was recorded individually using a 5-point Likert scale with options spanning from *very confident* to *not confident at all*.

The technology confidence score was calculated for each respondent. This score provides a summary of each respondent’s overall technology confidence based on the confidence response to each of the 4 technologies. The scores assigned for each response ranged from 0 (not confident at all) to 4 (very confident)*.* The confidence score for each respondent was the sum of the scores from their responses. The maximum possible confidence score was 16, and the minimum, 0. Cronbach α for the 4 confidence score items was .952.

#### By Job Role

Confidence responses were categorized by job role. Table 6 provides an overview of these results. *Very confident* was the most common response provided by respondents from all job roles, followed by *moderately confident*.

Table 7 highlights the mean confidence score calculated for each job role. The Kruskal–Wallis test indicated no significant difference (*P*=.64) between the social care worker and social worker confidence score.

Table 8 provides an overview of the mean confidence score by technology type. The maximum possible confidence score for any technology was 4 (very confident), and the minimum possible value was 0 (not confident at all). It can be observed that, on average, respondents were most confident with the use of smartphones, followed by desktop computers and laptops. Respondents expressed the least confidence in using tablets.

**Table 6:** Technology confidence responses versus job role.

Response	Job role			Overall, n (%)
	Social care worker, n (%)	Social worker, n (%)	Social work student, n (%)	
Not confident at all	60 (2.8)	12 (1.4)	2 (3.6)	74 (2.4)
Only slightly confident	116 (5.4)	32 (3.6)	3 (5.4)	151 (4.9)
Somewhat confident	213 (9.9)	92 (10.4)	2 (3.6)	307 (9.9)
Moderately confident	617 (28.6)	260 (29.4)	11 (19.6)	888 (28.7)
Very confident	1148 (53.3)	489 (55.3)	38 (67.9)	1675 (54.1)

**Table 7:** Mean technology confidence score versus job role.

Job role	Confidence score, mean (SD)
Social care worker	12.97 (3.83)
Social worker	13.32 (3.35)
Social work student	13.71 (3.95)
Overall	13.09 (3.70)

**Table 8:** Mean technology confidence score versus type of technological device

Type of technology	Confidence score, mean (SD)
Desktop computers	3.28 (0.97)
Laptops	3.27 (1.00)
Smartphones	3.30 (0.97)
Tablets	3.24 (1.02)

#### By Age

Confidence responses were also categorized by age group. Of note, responses from respondents who selected *prefer not to say* for age group were not included. Table 9 provides an overview of the responses from each age group. It was observed that the most common response is very confident for all age groups except for the ≥65 years group. There is a steady decline in the proportion of the very confident responses as age group increases and a general trend of an increase in less confident responses.

To further explore this trend, the mean confidence score was calculated for each age group. This is summarized in Table 10. It can be seen that the mean confidence score decreases as age group increases (r_s_=−0.314; *P*<.001).

**Table 9:** Technology confidence responses versus age group.

Age (years)	Technology confidence response				
	Not confident at all, n (%)	Only slightly confident, n (%)	Somewhat confident, n (%)	Moderately confident, n (%)	Very confident, n (%)
15-24	0 (0.0)	2 (0.8)	5 (2.0)	53 (21.7)	184 (75.4)
25-44	20 (1.5)	27 (2.0)	93 (7.0)	316 (23.8)	871 (65.6)
45-64	50 (3.4)	116 (7.8)	202 (13.6)	502 (33.9)	610 (41.2)
≥65	4 (14.3)	5 (17.9)	3 (10.7)	9 (32.1)	7 (25.0)

**Table 10:** Mean technology confidence scores versus age group.

Age (years)	Confidence score, mean (SD)
15-24	14.87 (1.94)
25-44	14.00 (3.12)
45-64	12.06 (4.04)
≥65	9.43 (5.13)

#### Confidence Versus Digital Skills

The relationship between digital skills and technology confidence was explored. A moderate positive correlation was identified (r_s_=0.482; *P*<.001), which indicates that higher self-reported digital skills levels are associated with high technology confidence.

### Learning and Development

#### Influencing Factors

Respondents were asked to state the factors that influence them to learn and develop. Table 11 provides an overview of the percentage of respondents who indicated each factor.

**Table 11:** Learning influencing factor versus job role.

Influencing factor	Job role			Overall, n (%)
	Social care worker, n (%)	Social worker, n (%)	Social work student, n (%)	
Future employment prospects	228 (42.3)	104 (46.8)	11 (78.6)	343 (44.3)
I want to develop my knowledge and skills	430 (79.8)	205 (92.3)	11 (78.6)	646 (83.4)
Obligation from employer	352 (65.3)	149 (67.1)	6 (42.9)	507 (65.4)
Obligation from regulating bodies	292 (54.2)	133 (60.0)	4 (28.6)	429 (55.4)
Other	6 (1.1)	7 (3.2)	0 (0.0)	13 (1.7)

#### Access

Respondents were asked to indicate the methods they used to access learning. Table 12 provides an overview of these results.

Respondents were provided with a list of e-learning tools and asked to state which of them they used to support learning and development at home and at work. Table 13 provides a comprehensive overview of the responses provided overall and by job role.

Table 14 provides an overview of the responses received regarding the usefulness of e-learning tools

Respondents were asked whether they would be interested in participating in e-learning and development delivered at home and at work. Table 15 presents the willingness to engage with e-learning tools by job role.

**Table 12:** Methods used to access learning versus job role.

Method of accessing learning	Job role			Overall, n (%)
	Social care worker, n (%)	Social worker, n (%)	Social work student, n (%)	
Face-to-face	444 (82.4)	192 (86.5)	13 (92.9)	649 (83.7)
e-learning	421 (78.1)	182 (82.0)	9 (64.3)	612 (79.0)
Reading information leaflets or workbooks	310 (57.5)	175 (78.8)	6 (42.9)	491 (63.4)
Other	25 (4.6)	26 (11.7)	1 (7.1)	52 (6.7)

**Table 13:** e-learning tools used at home and at work versus job role.

Type of technology, Location used		Job role			Overall, n (%)
		Social care worker, n (%)	Social worker, n (%)	Social work student, n (%)	
**Electronic books**					
	Home	195 (36.2)	108 (48.6)	10^a^ (71.4)	313^b^ (40.4)
	Work	111 (20.6)	66 (29.7)	6^a^ (42.9)	183^b^ (23.6)
**Mobile learning apps**					
	Home	276 (51.2)	106 (47.7)	8 (57.1)	390 (50.3)
	Work	133 (24.7)	57 (25.7)	7 (50.0)	197 (25.4)
**Online communities**					
	Home	221 (41.0)	81 (36.5)	5 (35.7)	307 (39.6)
	Work	126 (23.4)	58 (26.1)	5 (35.7)	189 (24.4)
**Others**					
	Home	44 (8.2)	9 (4.1)	1 (7.1)	54 (7.0)
	Work	18 (3.3)	4 (1.8)	1 (7.1)	23 (3.0)
**Podcasts**					
	Home	89 (16.5)	65 (29.3)	3 (21.4)	157 (20.3)
	Work	26 (4.8)	28 (12.6)	1 (7.1)	55 (7.1)
**Vlogs**					
	Home	67 (12.4)	26 (11.7)	2 (14.3)	95 (12.3)
	Work	17 (3.2)	6 (2.7)	0 (0.0)	23 (3.0)
**Websites**					
	Home	394 (73.1)	175 (78.8)	13 (92.9)	582 (75.1)
	Work	313 (58.1)	195 (87.8)	12 (85.7)	520 (67.1)

^a^Total n=14.

^b^Total n=775.

**Table 14:** Usefulness of e-learning tools versus job role.

Usefulness of e-learning tools	Job role				Overall, n (%)
	Social care worker, n (%)	Social worker, n (%)	Social work student, n (%)		
Extremely useful	112 (20.8)	40 (18.0)	6 (42.9)		158 (20.4)
Very useful	196 (36.4)	89 (40.1)	5 (35.7)		290 (37.4)
Somewhat useful	164 (30.4)	75 (33.8)	3 (21.4)		242 (31.2)
Not so useful	23 (4.3)	6 (2.7)	0 (0.0)		29 (3.7)
Not at all useful	7 (1.3)	3 (1.4)	0 (0.0)		10 (1.3)
I haven’t used them	37 (6.9)	9 (4.1)	0 (0.0)		46 (5.9)

**Table 15:** Willingness to engage with e-learning tools versus job role.

Willingness to engage with e-learning tools	Job role			Overall, n (%)	
	Social care worker, n (%)	Social worker, n (%)	Social work student, n (%)		
Yes, at home in my own time	337 (62.5)	116 (52.3)	11 (78.6)		464 (59.9)
Yes, at work	304 (56.4)	174 (78.4)	7 (50.0)		485 (62.6)
No, neither	54 (10.0)	21 (9.5)	2 (14.3)		77 (9.9)

#### The Value and Challenges of Technology Use for Learning

**Figure 1:**
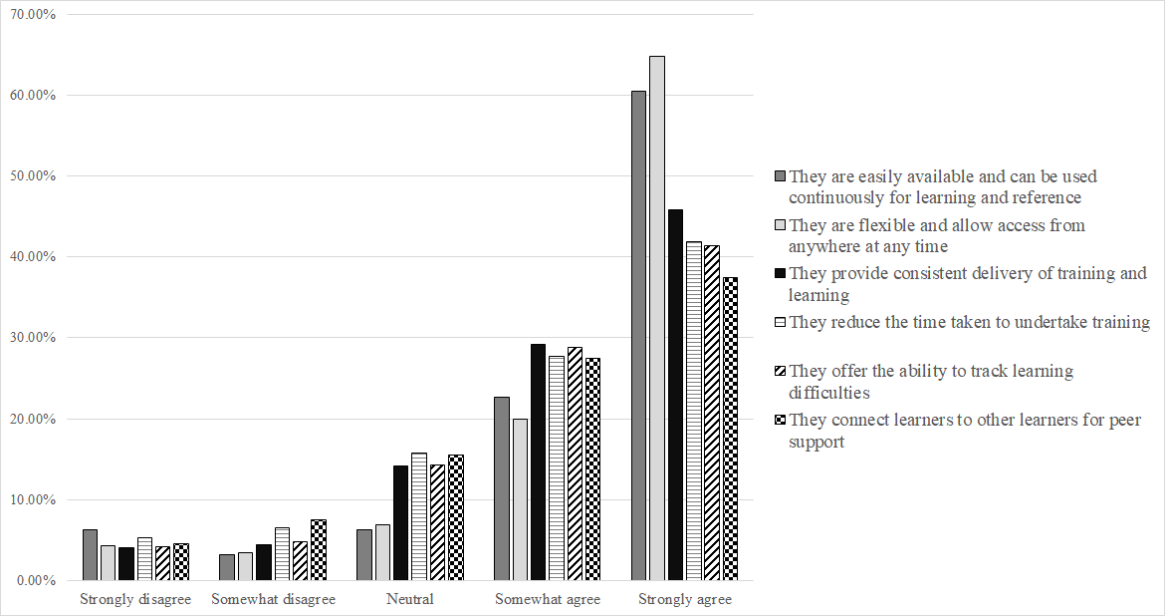
An overview of the level of agreement and disagreement to statements regarding the value of using technology for learning.

**Figure 2:**
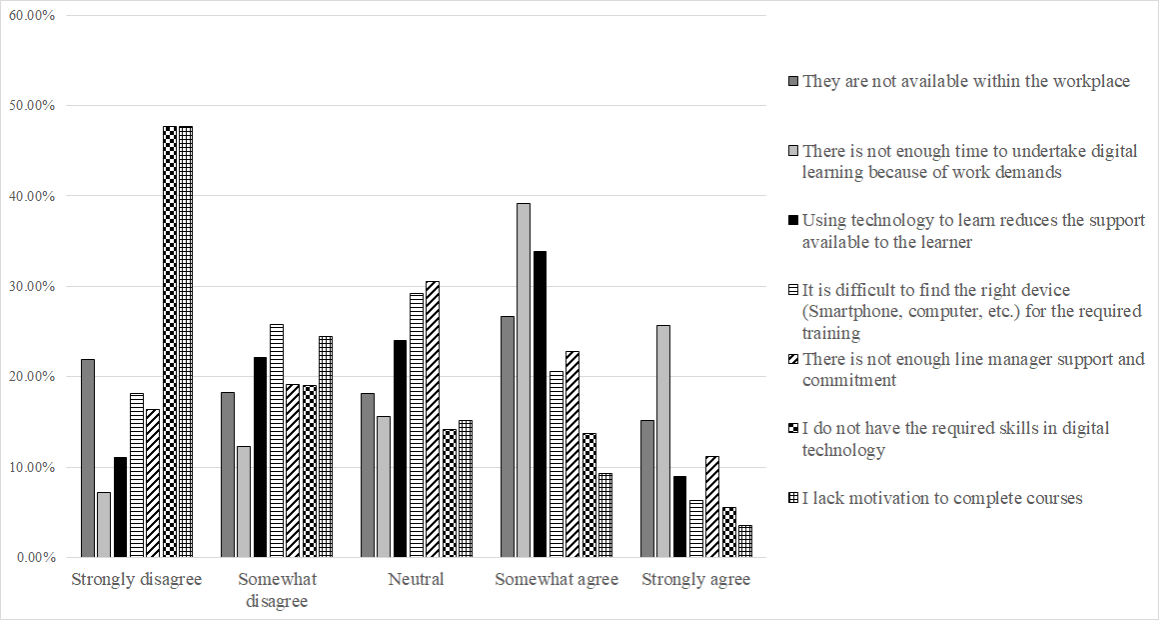
An overview of the level of agreement and disagreement to statements regarding the challenges of using technology for learning.

Respondents were asked to rate how strongly they agreed or disagreed with 6 positive statements about the value of technology to support learning and 7 statements regarding the challenges. Figure 1 summarizes the responses to the value statements, and Figure 2 summarizes the responses to the challenge statements. The majority of responses to statements regarding the benefits were positive. A total of 64.8% (502/775) of the respondents strongly agreed to the statement in relation to the flexibility of access from anywhere at any time. In addition, 60.5% (469/775) of the respondents strongly agreed that the technology is easily available and can be used continuously for learning and reference.

In terms of challenges, 64.9% (503/775) of the respondents at least somewhat agreed that there is not enough time to undertake digital learning because of work demands, and 42.8% (332/775) of the respondents at least somewhat agreed that the use of this technology to learn reduces the support available to the learner.

A technology value score and technology challenge score were calculated to summarize each respondent’s level of agreement or disagreement with the value and challenge statements. For each respondent, the scores were calculated by summing the values of the responses given to each of the respective statements. Values assigned to each response option ranged from −2 (strongly disagree) to 2 (strongly agree). The technology value score had a Cronbach α of .918. The maximum possible technology value score was 12 (strong agreement with all statements) and the minimum possible technology value score was −12 (strong disagreement with all statements). The technology challenge score had a Cronbach α of .766. The maximum possible technology challenge score was 14 (strong agreement with all statements) and the minimum possible technology challenge score was −14 (strong disagreement with all statements).

The mean technology value score was calculated for each job role. This is summarized in Table 16. It can be seen that the mean technology value score for all job roles was positive. There was a significant difference in the technology value score for each job role (*P*=.01).

The mean technology value score was also calculated for each age group. This is summarized in Table 17. Of note, responses from those who indicated age as *prefer not to say* were not included.

**Table 16:** Mean technology value score versus job role.

Job role	Technology value score, mean (SD)
Social care worker	7.2 (5.3)
Social worker	5.7 (6.1)
Social work student	6.6 (8.0)
Overall	6.8 (5.6)

**Table 17:** Mean technology value score versus age group.

Age (years)	Technology value score, mean (SD)
15-24	7.6 (4.2)
25-44	7.4 (5.1)
45-64	6.2 (6.2)
≥65	3.9 (6.0)

The mean technology value score for all age groups was positive. It can be seen that as age group increases, the mean technology value score decreases. There was a weak negative correlation between age and technology value score (r_s_=−0.088; *P*=.02).

The mean technology challenge score was calculated for each job role. This is summarized in Table 18. It can be seen that each job role had a negative mean technology challenge score. There was no significant difference in the technology challenge score for each job role (*P*=.79).

The mean technology challenge score was calculated for each age group. This is summarized in Table 19. It can be seen that each age group had a negative mean technology challenge score. This indicates that respondents within each age group slightly disagree with the challenges of technology use for learning.

A total of 9.9% (77/775) of the respondents indicated that they would not be willing to engage with e-learning tools at home or at work. Upon further analysis, it was revealed that these respondents had a mean digital skills score of 9.26 (SD 1.43), which is below the average digital skills score of 9.47 (SD 1.36). In addition, the mean confidence score for these participants was 11.61 (SD 4.48), which is below the overall mean confidence score of 13.09 (SD 3.70). The mean technology value score for these respondents was 4.79 (SD 5.86), which is below the overall mean technology value score of 6.8 (SD 5.6), and the mean technology challenge score for these respondents was 0.86 (SD 5.48), which is higher than the overall mean of 1.6 (5.5).

**Table 18:** Mean technology challenge score versus job role.

Job role	Technology challenge score, mean (SD)
Social care worker	−1.6 (5.7)
Social worker	−1.6 (5.3)
Social work student	−2.1 (3.2)
Overall	−1.6 (5.5)

**Table 19:** Mean technology challenge score versus age group.

Age (years)	Technology challenge score, mean (SD)
15-24	−1.84 (5.1)
25-44	−2.0 (5.5)
45-64	−1.3 (5.7)
≥65	−1.1 (4.2)

### Other Comments Regarding the Use of Technology for Learning and Development

Respondents were invited to provide further feedback regarding elements that may help or hinder them from using technology to support learning and development. Of the 131 additional comments that were provided, 28.2% (37/131) of comments mentioned that high workload or lack of time was a hindrance to engaging in training opportunities. Several respondents stated that they would like to have time ring-fenced to allow them to engage with digital learning opportunities.

## Discussion

### Digital Skills and Confidence

Respondents provided an overall high level of self-reported digital skills (mean digital skills score of 9.47, SD 1.36), with no significant difference in responses provided by respondents in each job role. The digital skills score was found to decrease as age group increased (r_s_=−0.262; *P*<.001); however, the oldest age group still demonstrated a high mean digital skills score of 7.86 (SD 3.02) out of a maximum possible score of 10. This is a very positive result, which indicates that the majority of respondents possess the core skills required to engage with digital learning and development solutions.

Technology confidence was again mostly positive, with 54.11% (1675/3095) of responses stating that they were very confident in their use of technology. This indicates that the majority of respondents felt confident in the use of the various platforms that would be suitable for deploying digital learning and development solutions. The same is true when analyzing by job role, with no significant difference (*P*=.64) in responses from each job role. This is a positive result; however, the results indicate key areas for focus. Particular focus should be given to members of the workforce within all job roles who indicated that they were only slightly confident or not confident at all in the use of technology. Overall, 2.39% (74/3095) of the respondents’ responses indicated that they were not confident at all in the use of a particular technology and 4.87% (151/3095) indicated that they were only slightly confident. There was a negative correlation between age group and confidence score. A total of 14% (4/28) of responses from respondents aged ≥65 years indicated that they were not confident at all in the use of some technologies. A total of 13.0% (101/775) of the respondents indicated that they would not be able to solve a problem with a digital device using web-based help, 9.0% (70/775) indicated that they would not be able to verify whether the web-based information they found was accurate, and 7.1% (55/775) could not buy or install apps on a device.

Although the majority of responses are positive, it is clear that there is a small portion of the workforce who would benefit from increased training in the use of technology. This is critical to ensure that every member of the workforce is able to benefit from the potential of digital learning and development and that a digital divide is not created. The results indicate that there is a positive correlation between self-reported digital skills and confidence score (r_s_=0.482; *P*<.001). As a result, it is recommended that members of the workforce who felt less confident are provided with the opportunity to engage with training sessions to increase their core digital skills. Comparison of the confidence score with the other metrics provided interesting results for consideration. It should be noted, however, that one limitation of this study is that the confidence score used in this survey is a novel score that has not been previously validated.

### Learning and Development

Encouraging results were received with regard to factors that influence respondents to learn and develop. “I want to develop my knowledge and skills” was the most popular response, selected by 83.4% (646/775) respondents. This suggests that respondents were motivated and have a genuine interest in learning and development, as it was a more popular response than *obligation from employer* (507/775, 65.4%) and *obligation from regulating bodies* (429/775, 55.4%). Interestingly, *future employment prospects* was the least popular option (343/775, 44.3%). This result indicates that a considerable portion of respondents are motivated to develop their knowledge and skills for reasons other than future employment prospects.

Face-to-face delivery was the most common method to access learning by all job roles. Although 83.7% (649/775) of the respondents accessed learning in this manner, this was closely followed by e-learning (612/775, 79.0%), which indicates that the majority of respondents were already engaging in informal methods of digital learning and development. This provides a promising foundation that can be further developed through formal provision of digital learning and development solutions. Websites were the most commonly used e-learning tools, followed by mobile learning apps. Interestingly, every e-learning tool was more commonly used at home than at work, which indicates that respondents are currently engaging in additional out-of-hours learning. The majority of respondents found e-learning tools to be very useful or extremely useful. This is encouraging, as these positive experiences with e-learning tools may translate to increased engagement with formal digital learning and development solutions. Nevertheless, a small number of respondents did not use these tools (46/775, 5.9%) or found them not so useful (29/775, 3.7%) or not at all useful (10/775, 1.3%). It would be beneficial to provide members of the workforce of this nature with an increased opportunity to engage with such tools and to further investigate why they did not find these tools useful.

Overall, the majority of respondents were willing to engage with e-learning tools at home or at work. Notably, 9.9% (77/775) of the respondents were not willing to engage with e-learning tools at home or at work.

The results indicate that the majority of respondents either somewhat or strongly agree with the value of using technology for learning and development. As the age group increases, the strength of agreement tends to decrease. Opinion on the challenges associated with technology for learning and development is further divided. The majority (517/775, 66.7%) of the respondents did not agree that they lacked the required skills in digital technology or that they lacked the motivation to complete courses (559/775, 72.1% at least somewhat disagree). This indicates a predominantly motivated workforce, the majority of which did not feel hindered by their level of skills in digital technology. Nevertheless, there is a clear benefit in offering additional digital skills training, as 19.2% (149/775) of the respondents at least somewhat agreed that they did not have the required skills in digital technology to facilitate learning and development. In addition, the majority (503/775, 64.9%) of the respondents at least somewhat agreed that they did not have enough time to undertake digital learning because of work demands.

### Maximizing Engagement With Digital Learning and Development Solutions

These results show that respondents who were not willing to engage with e-learning tools at home and at work were more likely to have lower self-reported digital skills, less technology confidence, see less value in technology for learning and development, and agree more with the challenges associated with technology for learning and development compared with the average respondent who was willing to engage with such tools. These findings suggest that offering training to increase digital skills and technology confidence, in addition to raising awareness of the benefits of the use of technology for learning and development, may increase the overall engagement with digital learning and development solutions.

### Conclusions

Continual development of the social care workforce is a key element in enabling better outcomes for the users of social care services. This work aims to identify the digital capability of the regulated social care workforce in Northern Ireland, in addition to exploring the workforce’s appetite for and barriers to using technology for learning and development. A total of 775 survey respondents facilitated the analysis of 127 metrics. The results indicated a workforce with an overall high level of self-reported basic digital skills and confidence. The results also highlighted a positive correlation between digital skills and technology confidence, a negative correlation between age and digital skill, and a negative correlation between age and perceived value of technology.

With regard to digital learning and development, the results also indicated a predominantly motivated workforce in which a considerable portion already engaged in informal e-learning. Reassuringly, respondents were more likely to be motivated to learn and develop through the desire to further develop their knowledge and skills rather than obligation from their employer or regulating bodies.

The results also indicated that lower self-reported basic digital skills and confidence were associated with less interest in engaging with e-learning tools and that a small portion of the workforce would benefit from additional basic digital skills training. These results provide clear areas of focus for the future use of technology for learning and development of the social care workforce.
